# COVID-19 and Intestinal Ischemia: A Multicenter Case Series

**DOI:** 10.3389/fmed.2022.879996

**Published:** 2022-05-18

**Authors:** Maryam Sarkardeh, Elahe Meftah, Narjes Mohammadzadeh, Javad Koushki, Zahra Sadrzadeh

**Affiliations:** ^1^Surgical Oncology Research Center, Faculty of Medicine, Mashhad University of Medical Sciences, Mashhad, Iran; ^2^Department of Surgery, Imam Reza Hospital, Mashhad University of Medical Sciences, Mashhad, Iran; ^3^Students' Scientific Research Center, Tehran University of Medical Sciences, Tehran, Iran; ^4^Department of Surgery, Imam Khomeini Hospital Complex, Tehran University of Medical Sciences, Tehran, Iran

**Keywords:** coronavirus disease 2019, COVID-19, SARS-CoV-2, bowel necrosis, mesenteric ischemia, intestinal perforation, case series

## Abstract

**Introduction:**

Gastrointestinal symptoms are common among COVID-19 patients. Although gastrointestinal involvements are mostly benign, they rarely indicate a severe pathology like intestinal ischemia. The present case series describes 21 patients with bowel ischemia, necrosis, or perforation.

**Methods:**

The present case series was conducted from April 2020 to February 2022 in the surgical wards of two Iranian hospitals. We retrospectively included adult patients with concomitant COVID-19 and intestinal ischemia. Primary outcomes were defined as the length of stay and survival.

**Results:**

Twenty-four patients with a median age of 61.5 years were included in the study. Sixteen (67%) patients were male, and 13 (54%) were without any comorbidities. Macrovascular mesenteric ischemia was not identified in 21 patients (87.5%). Gastrointestinal manifestations appeared on the median of seven days (range 2–21) after the diagnosis of COVID-19, with the most common symptom being abdominal pain. All the patients had a significantly elevated C-Reactive Protein prior to surgery, ranging from 68 to 362. D-dimer was measured in eight patients and was significantly elevated, ranging from 1,878 to over 5,000 ng/mL. One patient was managed conservatively due to a good clinical condition. Except for one patient with angioinvasive mucormycosis and one other with leukocytoclastic vasculitis, pathologic evaluation revealed general features of intestinal necrosis, including ulcer, hemorrhage, necrosis, neutrophilic infiltration (in seven patients), neutrophilic abscess (in four patients), and edema. Bowel necrosis accompanied mortality of 15 (62.5%) patients and a median of 6.5 days of hospital stay.

**Conclusion:**

Intestinal ischemia in COVID-19 patients is associated with a high mortality rate. Further research is needed to elucidate the dynamics of intestinal ischemia in the setting of COVID-19.

## Introduction

Coronavirus Disease 2019 (COVID-19) is primarily known to manifest with fever, cough, dyspnea, or other mild respiratory symptoms (1). However, several extra-pulmonary organ involvements have also been observed during the COVID-19 pandemic. The gastrointestinal tract is among the systems which are commonly affected by COVID-19. The most common gastrointestinal symptoms include nausea, vomiting, abdominal distress, and diarrhea. In the presence of gastrointestinal symptoms, a more severe COVID-19 is expected (2, 3). In some cases, gastrointestinal symptoms could indicate more severe pathologies, including intestinal ischemia. The reported prevalence of intestinal ischemia ranges from 0.7% in hospitalized patients with COVID-19 (4) to 10% among critically ill patients with COVID-19 (5). With a mortality of about 50%, intestinal ischemia in COVID-19 patients seems to be worth investigating (5, 6).

An association between COVID-19 and intestinal ischemia could be hypothesized based on the previous studies (4–12). Possible mechanisms include direct viral invasion of the intestinal and vascular epithelium via Angiotensin-Converting Enzyme 2 (ACE2) receptors (3), systemic expansion of pulmonary coagulopathy, complement-mediated vasculopathy (13–15), and platelet activation via binding of Spike protein to ACE2 receptor (16). However, the lack of knowledge in the field and the rarity of intestinal ischemia in the setting of COVID-19 profoundly limit our understanding of this possible association. The present case series was conducted to further clarify the characteristics of intestinal ischemia among the patients with COVID-19 infection.

## Materials and Methods

The present retrospective multicenter case series was conducted in two tertiary referral hospitals in Iran—Imam Khomeini Hospital Complex in Tehran and Imam Reza Hospital in Mashhad. This study describes patients with COVID-19 and intestinal ischemia admitted to the surgical ward of either of the two mentioned hospitals from April 2020 to February 2022. All the participants gave informed consent to take part in the study. They were assured of the confidentiality of their data and the equality of care for the participants and non-participants. A similar case series of four patients with intestinal necrosis from Imam Reza Hospital has been published previously (17). The present case series serves to further the knowledge derived from the previous study and discusses the possible etiologies of intestinal ischemia in the COVID-19 setting.

The inclusion criteria were as follows:

1. Aging 18 years or above
2. A positive reverse transcriptase-polymerase chain reaction (RT-PCR) for Severe Acute Respiratory Syndrome–Coronavirus–2 (SARS-CoV-2) from at least one nasopharyngeal sample
3. Respiratory signs and symptoms and COVID-19 diagnosis preceding gastrointestinal manifestations, or a concomitant diagnosis of COVID-19 and intestinal ischemia or perforation
4. Observing the evidence of intestinal ischemia or perforation in surgical exploration and imaging modalities (i.e., CT scan, sonography, chest x-ray, or abdominal x-ray)

Subjects were not included if they had any significant systemic conditions apart from COVID-19, which predisposed them to a hypercoagulable state. Patients with occlusion of the origin of superior mesenteric artery or vein were considered to have macrovascular involvement. Others with occlusions distal to the mentioned origins were defined as patients with microvascular involvement. Involvement of the origin of these large mesenteric vessels was evaluated through a CT scan or exploration of mesenteric vessels during the surgery.

Baseline characteristics of the patients and their laboratory findings prior to surgery were gathered from their medical records. The gathered information is listed in Tables 1, 2. The pattern of intestinal involvement, the received surgical care, and the outcome (length of stay and survival) were also gathered and summarized, as described in Table 3. If available, the plasma levels of LDH and D-dimer and the histopathologic reports of the excised specimens were gathered and summarized. Due to the small sample size, the results were reported with median (range).

**Table 1:** Baseline characteristics of the patients at the initiation of gastrointestinal manifestations.

	**Median (range)**	**Unit**
Age	61.5 (34–89)	years
Temperature, oral	37.15 (35.8–39.5)	0C
Pulse rate	110 (72–145)	per minute
Respiratory rate	19 (12–42)	per minute
O_2_ saturation	88 (70–96)	%
Systolic blood pressure	100 (75–144)	mmHg
Diastolic blood pressure	65 (40–95)	mmHg
WBC	17.45 (2.9–54.7)	x10^9^/Liter
Neutrophil	91 (72–96.7)	%
lymphocyte	5.8 (1.6–21)	%
Neutrophil to lymphocyte ratio	19.5 (3.43–60.44)	—
Hemoglobin	11 (6.7–14.4)	g/dL
Platelet	210 (37–540)	X10^3^/μL
Na	138.5 (130–156)	mEq/L
K	4.45 (3.1–6.1)	mEq/L
Urea	84 (26–261)	mg/dL
Creatinine	1.5 (0.5–7)	mg/dL
CRP	116 (68–362)	mg/dL
PT	15 (12–64)	Second
PTT	34.5 (24.3–96)	Second
INR	1.4 (1.1–6.4)	—
pH	7.33 (6.98–7.57)	—
PCO_2_	35.25 (21–56)	mmHg
HCO_3_	18.45 (6–30)	mEq/L
Base excess	−3.6 (−20–8)	mEq/L
Duration between onset of respiratory and gastrointestinal manifestations	7 (2–21)	Days
Length of hospital stay	6.5 (2–45)	Days

**Table 2:** Distribution of the characteristics of the patients.

		**Number of patients (of 21)**
Respiratory Symptom/Sign		
	Dyspnea	15
	Reduced oxygen saturation	14
	Tachypnea	8
	Cough	6
	Sore throat	4
Gastrointestinal Symptom/Sign		
	Abdominal pain	16
	Generalized tenderness	8
	Localized tenderness	6
	Failure to excrete gas and feces	5
	Distension	3
	Nausea and vomiting	3
	Rebound tenderness	2
	Guarding	2
	Hematochezia	2
	Asymptomatic	2
Comorbidities		
	Without comorbidities	13
	Hypertension	6
	Diabetes	4
	Congestive Heart Failure	2
	Atrial Fibrillation	2
	Coronary artery bypass graft	2
	Mitral valve replacement	2
	End-stage renal disease	1

**Table 3:** Progression and outcome of intestinal ischemia in the patients.

**Patient**	**Age (years)**	**Area of Involvement**	**Length of involvement (cm)**	**Pattern of involvement**	**Surgical management**	**Length of stay**	**Outcome**
1	58	ileum	140	ischemia	resection and end-to-end anastomosis	10	expired
2	51	ileum	40	necrosis and perforation in three sites	resection and end-to-end anastomosis	10	expired
3	60	ileum and colon	60 cm of ileum and full-length of colon	necrosis	resection and end-to-end anastomosis	6	expired
4	88	ileum	130	necrosis	resection and end-to-end anastomosis	5	expired
5	89	rectum	full-length	necrosis	abdominopelvic resection and Hartmann colostomy	3	expired
6	73	transverse colon	15	perforation in four sites	extended right colectomy and insertion of ileostomy	15	recovered
7	84	colon	full-length	ischemia	total colectomy and insertion of terminal ileostomy	2	expired
8	38	sigmoid	full-length	ischemia	sigmoid resection, terminal colostomy, and Hartmann procedure	15	recovered
9	33	jejunum	full-length	ischemia	resection and end-to-end anastomosis	25	recovered
10	52	small intestine	full-length	necrosis	none (due to the extent of necrosis)	2	expired
11	38	jejunum	5	perforation	resection and end-to-end anastomosis	45	recovered
12	67	sigmoid	full-length	perforation	sigmoidectomy and terminal Hartmann colostomy	5	expired
13	71	ileum	5	perforation	resection and end-to-end anastomosis	20	recovered
14	66	ileum	full-length	ischemia	resection of ileum and insertion of double-barrel ileostomy	16	expired
15	82	terminal ileum and small intestine	full-length	patchy ischemia of the small intestine and perforation of terminal ileum	resection of ileum and insertion of double-barrel ileostomy	20	expired
16	42	colon and terminal ileum	70 cm of terminal ileum and full-length of colorectal	ischemia and multiple perforations	primary enterorrhaphy	5	expired
17	63	terminal ileum	20	ischemia	resection and end-to-end anastomosis	12	recovered
18	34	jejunum	20	ischemia and perforations	resection and end-to-end anastomosis	43	expired
19	54	terminal ileum and ascending colon	20 cm of terminal ileum and full-length of ascending colon	necrosis	resection of terminal ileum and ascending colon and insertion of terminal ileostomy	2	expired
20	42	jejunum	15	Ischemia and perforation	primary enterorrhaphy	7	recovered
21	75	jejunum	50	ischemia	none (conservative management only)	6	recovered
22	82	jejunum, ileum, ascending and transverse colon	230	ischemia	embolectomy with Fogarty catheter	2	expired
23	88	jejunum	70	ischemia	resection and end-to-end anastomosis	4	expired
24	57	jejunum and ileum	90	ischemia	resection and end-to-end anastomosis	6	recovered

## Results

Twenty-four patients with a median age of 61.5 years (range 34–89) were included. Of these, 16 (67%) were male, and 13 (54%) were without any prior comorbidities. The most common comorbidities and respiratory symptoms were hypertension (25%) and dyspnea (625%), respectively. Except for six patients (25%), all the other patients were initially admitted to the intensive care unit (ICU) due to severe COVID-19. All the patients received the prophylactic dose of either unfractionated heparin or low molecular weight heparin. One patient was later switched to the therapeutic dose of unfractionated heparin since conservative management was favorable in his case. No one received anticoagulants before admission, except for two patients on warfarin due to a prosthetic mitral valve. Only three patients (numbers 20, 21, and 24) had received all three doses of the COVID-19 vaccine. One patient (number 22) had received two doses of the COVID-19 vaccine, and the rest were unvaccinated.

Three patients (12.5%) had leukopenia, three other patients (12.5%) had a normal leukocyte count, and 18 patients (75%) had leukocytosis with neutrophil dominancy. Only two patients (8%) had thrombocytosis. Thirteen (54%) had a normal platelet count, and 10 patients (42%) had thrombocytopenia. All patients had a high C-Reactive Protein (CRP) level, ranging from 68 to 362 (Table 2). D-dimer was measured in eight patients. Three of them had a D-dimer level of ≥5,000 ng/mL. D-dimer level of the five other patients was 4,502, 4,260, 3,534, 20,05, and 1,878 (Median = 4,381). LDH was measured in 13 patients and had a median (range) of 623 (250 – 4,338) units per liter.

All the patients had respiratory symptoms prior to gastrointestinal manifestation. The symptoms and signs of intestinal ischemia appeared on the median of seven days (range 2–21) after the initial respiratory symptoms. The most common symptom was abdominal pain. Two patients did not have any gastrointestinal symptoms or signs. Intestinal perforation of these two patients was suspected after observing a pneumoperitoneum in a chest CT scan. Abdominopelvic CT scan was the most common diagnostic modality (50%). The findings of the abdominopelvic CT scan were intestinal wall thickening (50%), mild to moderate free fluid (50%), pneumoperitoneum (25%), pneumatosis intestinalis (25%), and perforation (17%). Nine patients were hemodynamically unstable, five requiring vasopressors before surgery. Two of these patients received norepinephrine for longer than 5 days.

The ileum was the most common site of intestinal involvement affecting 10 patients (42%). Of these ten patients, four had the involvement of terminal ileum. Jejunum and colon were the next common sites of involvement, each being damaged in 33% of the patients. The extent of colonic involvement was full-length in two patients (8%), sigmoid in two (8%), transverse colon in two (8%), and ascending colon in two patient (8%). Two patients had full-length involvement of the small intestine and one had rectal involvement (Table 3). Three patients were categorized as having macrovascular involvement (numbers 22–24).

Resection and end-to-end anastomosis of the small intestine was the most common surgical intervention (46%). A terminal ileostomy was performed for three patients, a Hartmann colostomy was performed for three others, and a double-barrel ileostomy for two patients. Primary enterorrhaphy was performed for two patients. Embolectomy with Fogarty catheter was performed for one patient with superior mesenteric artery thrombosis. One patient (number 10) did not receive any surgical repair due to the extent of necrosis. One other patient (number 21) was managed medically due to stable condition and the absence of signs of necrosis or perforation in the CT scan. Additionally, the patient did not have any symptoms of peritoneal irritation. The patient experienced an uncomplicated recovery and was symptom-free in the follow-up.

**Figure 1:**
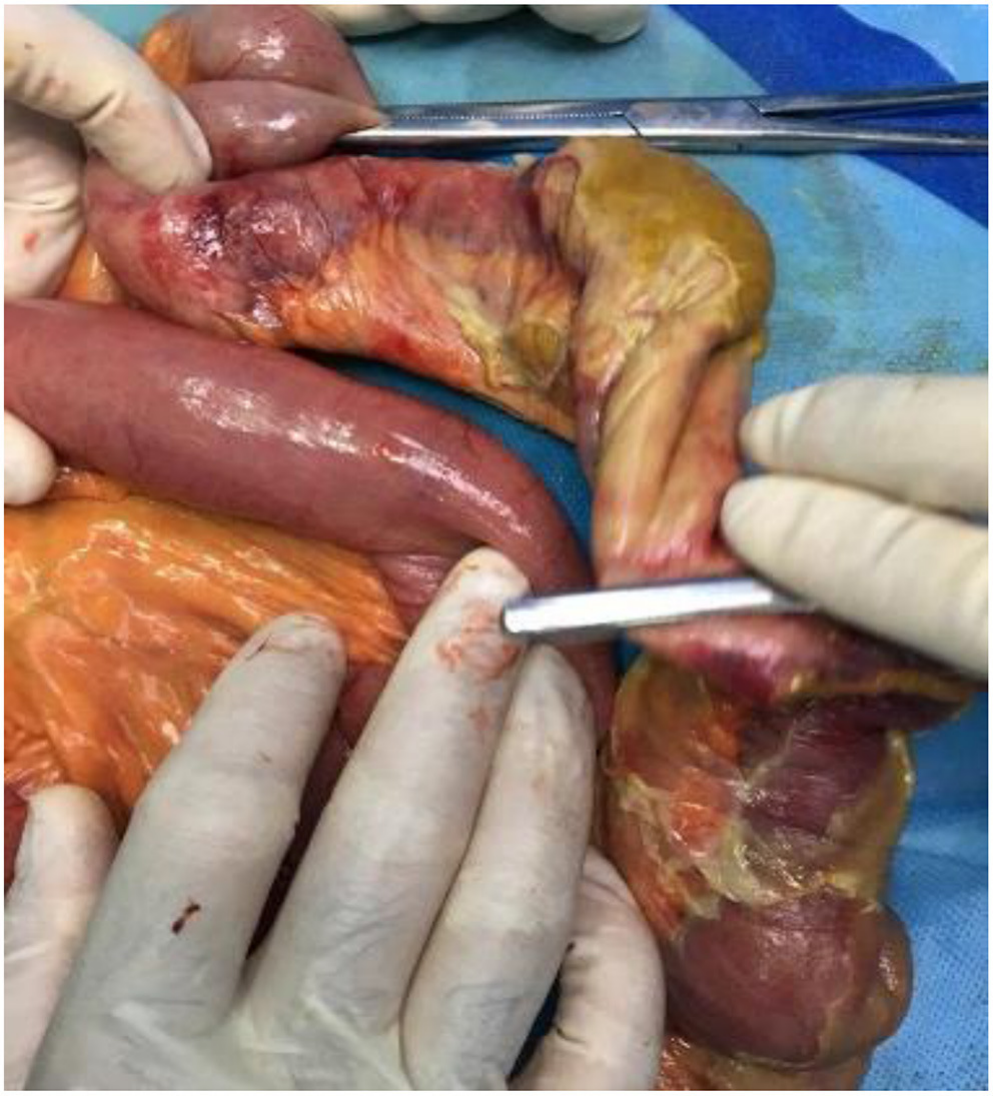
Yellow appearance of the necrotic intestine found in the gross examination (17).

Sixteen patients had histopathologic evaluations. One patient had leukocytoclastic vasculitis on microscopic examination, and one other had angioinvasive mucormycosis. Angioinvasive mucormycosis was found in a diabetic patient (number 19) and had resulted in necrosis of ascending colon and 20 cm of the terminal ileum. The patient with leukocytoclastic vasculitis (number 6) did not have any prior comorbidity and was initially diagnosed based on the histopathologic features of skin lesions. Except for the two mentioned cases, histopathologic examination revealed general features, including necrosis, ulcer, hemorrhage (in nine patients), neutrophilic infiltration (in seven patients) or abscess (in four patients), and edema. Fibrin microthrombi and severe vascular congestion were observed in six and three patients, respectively. Vascular pathologies were not noted in two of the patients. Necrosis was evident in 12 cases, of which nine were transmural. Four patients had coagulative and liquefactive necrosis, and atrophic mucosa was present in eight. Five patients had yellow ischemia or necrosis (Figure 1), one with saponification features.

Mortality occurred in 15 patients (62.5%). Multi-organ failure due to septic shock was the most common cause of death (observed in eight patients), followed by respiratory arrest (in five) and cardiac arrest (in two patients). The median length of hospital stay was 15 days among survivors (range 6 to 45) and 5 days among non-survivors (range 2 to 43). The overall median length of stay was 6.5 days.

## Discussion

The present study highlights a possible association between COVID-19 and intestinal ischemia. With a 62.5% mortality rate observed in this study, clinical suspicion could be the key to preventing death from intestinal ischemia.

The present literature lacks a precise explanation for the pathophysiology of intestinal ischemia in the setting of COVID-19. The high enterocytic concentration of the ACE2 receptor, the receptor through which SARS-CoV-2 invades the cells, could predispose the intestinal tract to direct viral invasion and damage (3). Similar to a case report by Jacob et al. (11), two of our patients did not have any vascular abnormality on histopathologic examinations. We hypothesize that direct viral invasion of the intestinal wall through enterocytic ACE2 receptors could have caused intestinal ischemia in the two mentioned cases. ACE2 receptors are mainly concentrated in the ileum and colon (14), the two common sites of involvement in our patients (observed in three-fourths of the patients). The mentioned finding further supports the hypothesis regarding the role of enterocytic ACE2 receptors in the pathogenesis of intestinal ischemia.

In patients with patency of the large mesenteric vessels, microvascular pathology was considered the most probable etiology of intestinal ischemia. Intestinal ischemia could be attributed to the hypercoagulable state secondary to COVID-19. Although any critical illness may result in hypercoagulability (15), the hypercoagulability in the setting of COVID-19 is thought to depend upon other additional factors, which will be discussed below. An indicator of a novel mechanism of ischemia could be the specific manifestation of the necrotic intestine in some COVID-19 patients. Five of our patients had a yellow appearance of ischemia or necrosis (Figure 1), similar to the previous reports and contrary to the typically-observed black or purple discoloration of the necrotic area (12, 18). One patient had saponification necrosis, and four had concomitant coagulative and liquefactive necrosis.

Extra-pulmonary organ damage is thought to be secondary to endothelial damage and systemic microangiopathy. Healthy endothelium is an immunomodulatory barrier against viral infections, maintains hemostasis, and aids vascular patency through nitric oxide production (13, 14). The damaged endothelium activates the cascade of thrombosis, augments thrombosis formation by releasing procoagulant cytokines, and inhibits thrombolysis by releasing plasminogen activator inhibitor. Additionally, the disruption of endothelial integrity and release of inflammatory cytokines allows for further leukocyte migration and aggravation of the localized inflammation (14). The suggested pathophysiologies of endothelial damage and microangiopathy are the systemic expansion of pulmonary coagulopathy, direct vascular damage through endothelial ACE2 receptors, and complement-mediated vasculopathy (13–15). Complement-mediated vasculopathy is part of an inflammatory process involving various substances like tissue factor, von Willebrand factor, and Mannan-binding lectin serine protease 2 that leads to immunothrombosis in COVID-19 (14). The neutrophil extracellular trap is another suggested etiology of COVID-19 immunothrombosis. Based on the role of neutrophils in immunothrombosis, the prognostic value of leukocytosis with an increased absolute neutrophil count, increased neutrophil to lymphocyte ratio, and even lymphocytopenia is justifiable (14, 15). All the mentioned changes in hematologic indices were present in the patients involved in this study. Another reason for hypercoagulability in COVID-19 may be platelet invasion through ACE2 receptors, thereby resulting in platelet hyperactivation, platelet aggregation, the release of platelet granules, and thrombus formation (16).

According to the findings of the present study, an uncommon etiology of intestinal necrosis could be angioinvasive mucormycosis. Gastrointestinal necrosis due to mucormycosis has been mentioned as a rare sequela of COVID-19 (19–21). Gastrointestinal involvement is among the rare presentations of mucormycosis, with the stomach and intestine as the most commonly involved organs, respectively (19). Two significant risk factors are diabetes and corticosteroid therapy (19), both present in one of our patients. Another extremely rare etiology of intestinal ischemia observed in the present study was leukocytoclastic vasculitis (LCV), a small vessel vasculitis with microvascular immune complex deposition mainly resulting in skin lesions. LCV is mainly idiopathic (22), although it could be secondary to an underlying infection, drugs, malignancy, inflammation, or rheumatologic cause (23, 24). Based on the previous studies, LCV secondary to COVID-19 infection is a rare phenomenon which is mainly limited to the skin (23). There are few case reports of non-COVID-19 cases with gastrointestinal involvement of LCV (22, 24). However, literature is lacking on the LCV-induced intestinal ischemia among COVID-19 patients. Camprodon Gomez et al. describe a case of LCV with gastrointestinal involvement. The patient developed palpable purpura, abdominal pain, diarrhea, and hematochezia one month after the diagnosis of COVID-19. As no intestinal sample was taken nor any surgical exploration was performed, evaluating the presence of mural necrosis in the described patient was not feasible (23). A similar phenomenon—Henoch Schonlien Purpura—was reported to severely affect the gastrointestinal tract in a COVID-19 patient (25).

Most of our patients were without any comorbidities, contrary to the majority of the previous studies. The symptoms and signs of intestinal ischemia were initiated 7 days following the respiratory manifestations of COVID-19. The precedence of respiratory manifestations has also been mentioned in the previous studies (6, 7, 10, 11, 26). However, some studies report the concomitant PCR confirmation of COVID-19 and intestinal ischemia (7, 8, 12). Although most patients presented with gastrointestinal manifestations, two patients were not suspected of intestinal ischemia until imaging studies were ordered. In line with this observation, Correa Neto et al. describe a patient without any gastrointestinal symptoms whose intestinal perforation was diagnosed only after observing an extensive pneumoperitoneum in chest x-ray (8).

Compared to the patients without intestinal ischemia from the same hospital (1), CRP was significantly higher among the patients in the present study. Increased CRP is also reported in the previous studies (5, 11, 26) and is known as a marker of poor prognosis among COVID-19 patients (27, 28). Similar to the previous studies (7, 8), D-dimer was also significantly increased among our patients, surpassing 5,000 ng/mL in some patients. D-dimer is a predictive factor of disease severity and mortality among COVID-19 patients (8, 13, 27, 28) and is also suggestive of thrombotic microangiopathy (13, 14). The mentioned findings are aligned with the hypothetical role of inflammatory markers in predicting a hypercoagulable state in COVID-19 (15). It is noteworthy that a normal CRP and D-dimer may not necessarily rule out intestinal ischemia. Soeselo et al. describe a patient with gastrointestinal manifestations and normal CRP and D-dimer, diagnosed with intestinal necrosis and perforation (10). Based on what was mentioned, the predictive value of CRP and D-dimer are best interpreted based on the clinical presentations.

In the present study, the mortality of intestinal ischemia in COVID-19 was 62.5%. The mentioned rate was measured in a sample with unvaccinated individuals as the majority. Three patients had received all three doses of the COVID-19 vaccine, one patient had received two doses, and the rest were unvaccinated. According to the significant effect of three doses of the COVID-19 vaccine on reducing mortality (29), the mentioned mortality rate could have been much lower if measured in a fully vaccinated population. All patients who received three doses of vaccine survived. However, mortality occurred in the patient who had received two doses of the COVID-19 vaccine. The high mortality rate in this study could also be partially due to the factors of poor prognosis mentioned above (15, 28). However, their independence of intestinal ischemia is not apparent. The diagnosis of intestinal ischemia in COVID-19 patients with gastrointestinal manifestations should be kept in mind (4), as this condition accompanies approximately 50% chance of mortality (5, 6) or even about two-thirds, as observed in this study. Additionally, the length of hospital stay seems to be affected by intestinal ischemia. The median length of stay was 15 days among survivors, significantly prolonged compared to non-survivors. The same centers of the study population have reported a median of 8 days (1) and 5 days (30) of stay, both significantly lower than the length of stay of the survivors. The high mortality and prolonged length of stay necessitate clinicians' suspicion and attention to intestinal ischemia to prevent morbidity and mortality.

### Strengths and Limitations

Among the strengths of the present study are the multicenter conduction, the relatively large sample compared to most of the previous case series, and the discussion of rare etiologies for COVID-19-induced intestinal ischemia. Some limitations included the unavailability of the histopathology report, LDH, and D-dimer for some patients. Lacking vascular evaluation with CT angiography was also among the limitations of our study. Next of kin refusal of autopsy limited the evaluation of any possible systemic condition related to intestinal ischemia. Additionally, the retrospective and observational nature of the study limited the extraction of a causal relationship and predisposed the study to selection bias. Despite all the mentioned limitations, the present study sheds light on the possible association of COVID-19 and intestinal ischemia and suggests some clinical clues in favor of the explained condition.

## Conclusion

Intestinal ischemia is one of the rare presentations of COVID-19 infection. Intestinal ischemia could occur secondarily to the infection or due to its complications. Vasculitis and mucormycosis are among the COVID-19 complications resulting in bowel ischemia and necrosis as an extremely rare presentation. Although bowel necrosis among COVID-19 patients is rare, its association with high mortality rates and prolonged length of stay necessitates clinical suspicion and prompt intervention.

## Data Availability Statement

The raw data supporting the conclusions of this article will be made available by the authors, without undue reservation.

## Ethics Statement

Ethical review and approval was not required for the study on human participants in accordance with the local legislation and institutional requirements. The patients/participants provided their written informed consent to participate in this study.

## Author Contributions

NM and MS performed the surgeries and designed the study. NM, MS, JK, and ZS gathered the relevant data. EM wrote the initial version of the manuscript. All the authors drafted and revised the final version of the manuscript.

## Conflict of Interest

The authors declare that the research was conducted in the absence of any commercial or financial relationships that could be construed as a potential conflict of interest.

## Publisher's Note

All claims expressed in this article are solely those of the authors and do not necessarily represent those of their affiliated organizations, or those of the publisher, the editors and the reviewers. Any product that may be evaluated in this article, or claim that may be made by its manufacturer, is not guaranteed or endorsed by the publisher.
